# Green Synthesis and Investigation of Surface Effects of α-Fe_2_O_3_@TiO_2_ Nanocomposites by Impedance Spectroscopy

**DOI:** 10.3390/ma15165768

**Published:** 2022-08-21

**Authors:** Hira Sultan, Aeysha Sultan, Raha Orfali, Shagufta Perveen, Tahir Ali, Sana Ullah, Haji Muhammad Anas, Safina Ghaffar, Areej Al-Taweel, Muhammad Waqas, Waseem Shahzad, Aftaab Kareem, Aqsa Liaqat, Zaman Ashraf, Ayesha Shahid, Abdul Rauf

**Affiliations:** 1 MFMG, Physics Division, PINSTECH, P.O. Box Nilore, Islamabad 44000, Pakistan; 2 Department of Chemistry, Division of Science & Technology, University of Education, Lahore 54700, Pakistan; 3 Department of Pharmacognosy, Collage of Pharmacy, King Saud University, P.O. Box 2457, Ryiadh 11451, Saudi Arabia; 4 Department of Chemistry, School of Computer, Mathematical and Natural Sciences, Morgan State University, Baltimore, MD 21251, USA

**Keywords:** surface effects, green synthesis, impedance spectroscopy, hematite, ginger extract, iron oxide-titania nanocomposite

## Abstract

Nanocomposites based on iron oxide/titanium oxide nanoparticles were prepared by employing green synthesis, which involved phytochemical-mediated reduction using ginger extract. XRD confirmed the composite formation, while scanning electron microscopy (SEM), dynamic light scattering (DLS), and energy-dispersive X-ray spectroscopy (EDX) was employed to investigate the particle size, particle morphology, and elemental analysis. SEM indicated the formation of particles with non-uniform shape and size distribution, while EDX confirmed the presence of Fe, Ti and oxygen in their elemental state. The surface effects were investigated by Fourier transform infrared radiation (FTIR) and impedance spectroscopy (IS) at room temperature. IS confirmed the co-existence of grains and grain boundaries. Thus, FTIR and IS analysis helped establish a correlation between enhanced surface activity and the synthesis route adopted. It was established that the surface activity was sensitive to the synthesis route adopted. The sample density, variation in grain size, and electrical resistivity were linked with surface defects, and these defects were related to temperature. The disorder and defects created trap centers at the sample’s surface, leading to adsorption of CO_2_ from the environment.

## 1. Introduction

TiO_2_ is a popular semiconductor that has gained the attention of scientists for various applications, i.e., as a supercapacitor, photocatalyst, and environment purifier [1]. TiO_2_ naturally occurs in three polymorphs: anatase, rutile, and brookite [2,3]. Due to the high photocatalytic activity and high dielectric constant, rutile form has been widely studied; however, it has a wide bandgap of 3.02 eV with a high electron-hole recombination rate, limiting its applications [4,5]. Owing to this limitation, researchers are working on the composite formation and doping of TiO_2_ with other metal oxides to tune this bandgap [6]. Fe_2_O_3_ has a comparable bandgap of 2.2 eV, with a half-filled d orbital and a similarly ionic radius, making it a potential candidate for this purpose [7]. It is reported that Fe^3+^ adds an extra energy level in the TiO_2_ bandgap, generating an intermediate level for photoelectrons to reside in, thus reducing the electron-hole recombination and improving the conduction properties [7,8]. It is also important to note that the synthesis route has direct implications on structural and electromagnetic properties [9]. Researchers have reported that the direct impregnation of Fe_2_O_3_ on TiO_2_ showed decreased photocatalytic performance, more than was expected from a conventionally synthesized α-Fe_2_O_3_@TiO_2_ nanocomposite [10]. Fe_2_O_3_ is oxygen deficient, especially when treated at low temperatures, creating oxygen vacancies that act as adsorption sites for O_2_ and CO_2._ Thus, the sample surface is adsorbed by environmental O_2_ and CO_2_ either via chemisorption or physisorption [11].

Owing to ever-growing pollution and energy shortages, recent trends involve the utilization of green synthetic protocols [12]. A much newer approach is soft synthesis, which includes solid-state synthesis methods that involve minimal use of high temperature and pressure conditions. Nanoparticle synthesis can be achieved at sintering temperatures as low as 150 °C. Such methods are economically feasible, and they are expected to be the future of cheap and reliable synthesis. The precursor polynuclear multimetallic compounds yield nanostructures upon decomposition [13]. Green chemistry relies on using natural ingredients such as extracts from plants’ leaves, flowers, roots, seeds, yeast, fungi, and bacteria as an alternative to the hazardous chemicals used in conventional synthesis methods [14]. Consequently, it offers the possibility of preparing nanostructured magnetic ferrites [15,16,17,18].

In the present work, ginger extract is used as it contains biomolecules and metabolites [19,20,21,22]. These metabolites can act as capping agents, reducing agents, and stabilizing and/or chelating agents, which can influence the size, the shape, and the morphology of the nanoparticles [17,18]. However, one of the main setbacks of such methods is the introduction of defects and disorder in the sample. Despite being a cheap and inexpensive method, heterogeneities, i.e., porosity and impurities, are expected to be introduced into the system due to low-temperature sintering; these are efficiently eliminated with high-temperature heat treatment [23]. Low-temperature sintering for small durations leads to the formation of particles with non-homogeneous particle-size distribution and structural defects [24]. Such structural defects on the sample surface are reactive towards environmental gases. The electrochemical response of a sample is greatly affected by surface heterogeneity and surface roughness [25]. Constant phase elements give systems a quantitative measure of heterogeneity [26]. In this article, the prime focus will be the study of heterogeneity introduced in the sample prepared from green chemistry. The effects of oxygen reduction reactions (ORR) and porosity on the reactive surface in the sample will be studied via various experimental techniques

The present work is based on the impedance response of various heterogeneities (surface effects, electrode effects, and surface diffusion) resulting from the green chemistry synthesis of nanocomposites of α-Fe_2_O_3_@TiO_2_. The composites were prepared using ginger root extract mediated wet ferritization, followed by in situ decomposition. The synthesized composites were subjected to electrochemical impedance spectroscopy studies. Correlations between the structural and impedance characteristics of the α-Fe_2_O_3_@TiO_2_ nanocomposites were established.

## 2. Materials and Methods

All reagents used were of analytical grade and were used without further purification. Ginger roots were purchased from the local market in Faisalabad, Punjab, Pakistan.

### 2.1. Preparation of Extracts

5 g of roots (ginger) were ground with a mortar and pestle. The ground material was placed in 400 mL of distilled water, and the mixture was boiled and stirred until the volume reduced to half. The beige color extract (pH: 4) was cooled to the ambient temperature and filtered under reduced pressure to yield the extract.

### 2.2. Synthesis of Ferrite Composite

**Figure 1:**
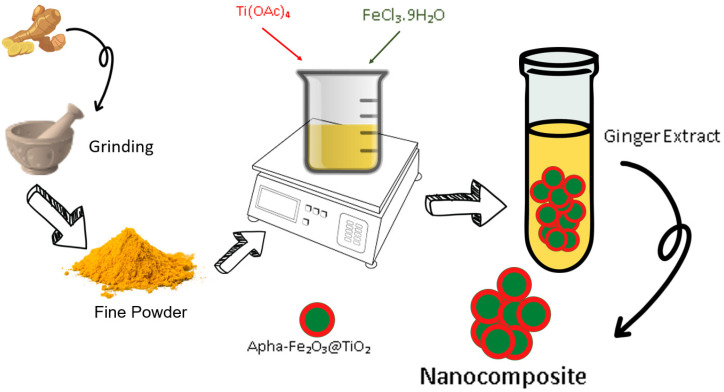
Schematic diagram of the green synthesis of α-Fe_2_O_3_@TiO_2_ nanocomposites.

The metal salts in the stoichiometric ratio (Fe^3+/^Ti^4+^, 2:1) were gradually added to the aqueous extracts of ginger extract (200 mL) under constant stirring. The resulting solution was concentrated on a magnetic hot plate. Initially, the solution turned into a gel, followed by the formation of semi-solids upon in situ decomposition. Upon further stirring and heating, the semi-solid turned into a fine solid powder. During the whole procedure, drastic changes in color and weight were observed. The solid powder was heated until no further reduction in weight was observed. The powdered ferrite composites were pressed into pellets under a pressure of 5 MPa, and sintered for 2 h at 150 °C. The schematic diagram is shown in Figure 1. 

## 3. Characterization of Prepared Compounds

A SIEMENS D5000 diffractometer was utilized for X-ray diffraction (XRD) data collection. Data was collected for a 2θ range of 20° to 80° using Cu-Kα radiations (λ = 1.54026 Å) with a step size of 0.05°, and a scan rate of 2.0 s/step. The surface morphology of the as-synthesized samples was investigated using SEM (Tescan MAIA3, Cardiff, UK), and the compositional analysis was carried by an Oxford instrument EDX detector. DLS (Litesizer 500 BM10, Anton Paar, Ukraine) was used to measure the average particle size. FTIR (Nicolet iS50 spectrometer, Waltham, MA, USA) was used to detect various bond stretching in the sample. Impedance studies were carried out using an Alpha-N Analyser (Novocontrol Germany) in the frequency spectrum range of 1.0 to 5 × 10^6^ Hz; the electrode contacts were made with copper wire using silver paints as adhesive material. An AC voltage of 0.2 V was applied to the sample for these studies. WINDETA and ZView software were used for data acquisition and fitting results.

## 4. Results

### 4.1. XRD

**Figure 2:**
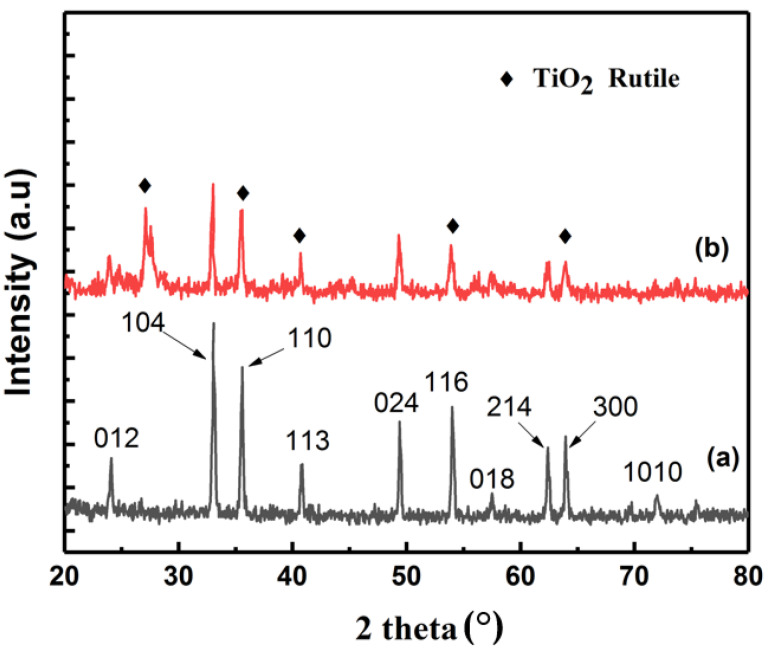
(**a**) XRD pattern for pristine α-Fe_2_O_3_ hematite powder. (**b**) XRD pattern of green synthesized α-Fe_2_O_3_@ TiO_2_.

Figure 2 shows the XRD pattern of the green synthesized α-Fe_2_O_3_@TiO_2_ nanocomposite. The XRD pattern confirms the formation of the α-Fe_2_O_3_@TiO_2_ nanocomposite. The main diffraction peaks corresponding to hematite planes (012), (104), (110), (113), (024), (116), (018), (214), (300), (1010), and (220), which matched with the XRD pattern of pristine α-Fe_2_O_3_ powder, were present. This XRD pattern is consistent with that reported by Vasiljevic et al. [27]. The additional peaks of the TiO_2_ corresponding to rutile phase are indicated in Figure 2. The dominant planes corresponding to rutile phase are (110), (101), (111), (211), and (002). The dominance of rutile form was also reported by Vasiljevic et al.; according to the authors, the presence of hematite lowers the temperature for the conversion of anatase to rutile form, thus making rutile polymorph the dominant form [27]. It was found that α-Fe_2_O_3_ (hematite), having R3c symmetry and TiO_2_ rutile phase with P 42/mnm geometry, was present in the composite as indicated by the XRD peaks [28].

### 4.2. SEM

**Figure 3:**
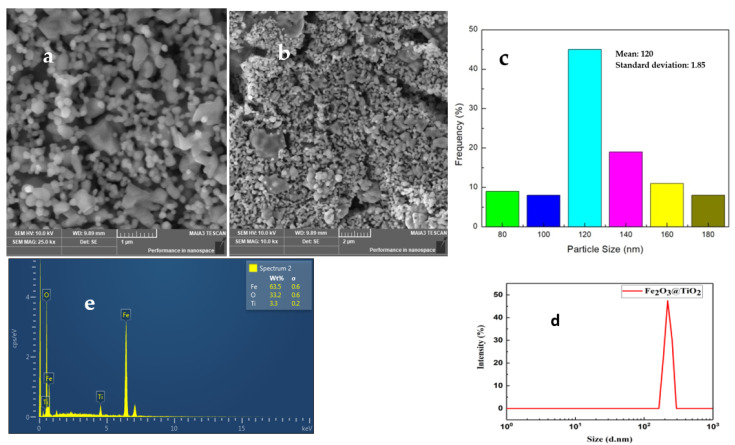
(**a**,**b**) SEM; (**c**) particle size distribution; (**d**) particle size distribution plot for α-Fe_2_O_3_@TiO_2_; (**e**) EDX image of α-Fe_2_O_3_@ TiO_2_ nanocomposite.

The morphological and compositional analysis of the α-Fe_2_O_3_@TiO_2_ nanocomposites was carried out by field emission scanning electron microscopy (FESEM) and the related energy dispersive X-ray spectroscopy (EDX). The SEM micrographs of the material at various magnifications are shown in Figure 3. The micrographs show uniformly distributed spherical particles along with the formation of some agglomerates of irregular shape. Careful particle size analysis indicated a particle size distribution in the range of 80 to 200 nm [29].

The particle size distribution obtained from SEM, shown in Figure 3c, indicated the majority of the particles were in the range of 120 to 140 nm. Litesizer 500 BM10 was also used for the dynamic light scattering analysis to determine the particle size in the nanocomposite. The sample powder was dispersed in water at room temperature. The mean intensity was 394.86 kcps for 15 runs. The particle size was found to be in the range of 100–400 nm as shown in Figure 3d. The reason for the discrepancy in the particle size determined by SEM and dynamic light scattering analysis may have arisen from the fact that agglomerated particles with larger sizes were not included in the SEM analysis.

The composition of the nanocomposite was verified by the EDX analysis. The EDX spectrum is shown in Figure 3e, with the corresponding elemental composition in wt%. The presence of Fe, O, and Ti corroborated the XRD results and indicated the formation of α-Fe_2_O_3_@TiO_2_, while the composition of Fe and O was in line with the Fe_2_O_3_ [30]. No significant difference in the chemical composition of the spherical and agglomerated particles was observed.

Table 1 represents the quantitative elemental composition of the sample as indicated by EDX (Figure 3e)

### 4.3. DLS

Litesizer 500 BM10 was used for the Dynamic light scattering analysis to determine the particle size in the nanocomposite. The sample powder was dispersed in water at room temperature. The mean intensity was 394.86 kcps for 15 runs. The particle size was found to be in the range of 100–400 nm as shown in Figure 3d. The difference in particle size between the particle analyser and SEM suggests possible agglomeration in the nanoparticles (Figure 3a,b).

### 4.4. FTIR

**Figure 4:**
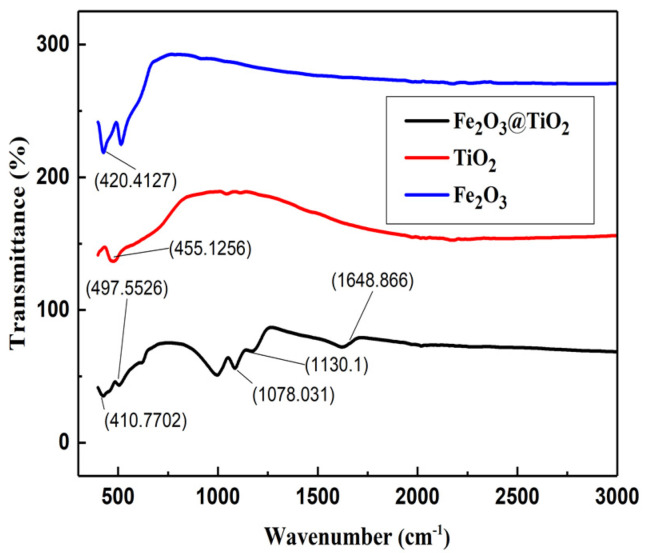
FTIR plot for α-Fe_2_O_3_@TiO_2_ nanocomposites (**blue**) TiO_2_ result pristine powder (**red**) Fe_2_O_3_ pristine powder (**black**).

The FTIR of the synthesized material was observed in comparison with the pristine powder of TiO_2_ and Fe_2_O_3_. The obtained data was in the range of 350 to 2000 cm^−1^ as shown in Figure 4.

In the pattern for the α-Fe_2_O_3_@TiO_2_ nanocomposite, the peak at 410 cm^−^^1^ corresponds to Fe–O stretching mode, while the peak observed at 497 cm^−^^1^ is due to the Ti–O stretching mode; these peaks are shifted to 420 cm^−^^1^ and 455 cm^−^^1^ for Fe–O and Ti–O stretching modes in the case of α-Fe_2_O_3_@TiO_2_ nanocomposite [31]. The peaks observed at 1078 cm^−^^1^ contributed to symmetrical CO_3_^2^^−^ anion mode stretching [32]. The peak at 1130 cm^−^^1^ corresponds to C–O stretching vibrations. It suggests the possibility of the adsorption of CO_2_(g) from the environment on the reactive sample surface, forming a layer of carbonate, as indicated by Equation (1) [33,34,35,36]. The broad peak appearing at 1648 cm^−^^1^ corresponds to the formation of carbonate species [31,32]. The FTIR spectrum shows the presence of chemical heterogeneities in α-Fe_2_O_3_@TiO_2_ nanocomposite.

CO_2_(g) + O^2−^(lattice) → CO_3_^2−^ (1)

### 4.5. Impedance Spectroscopy

**Figure 5:**
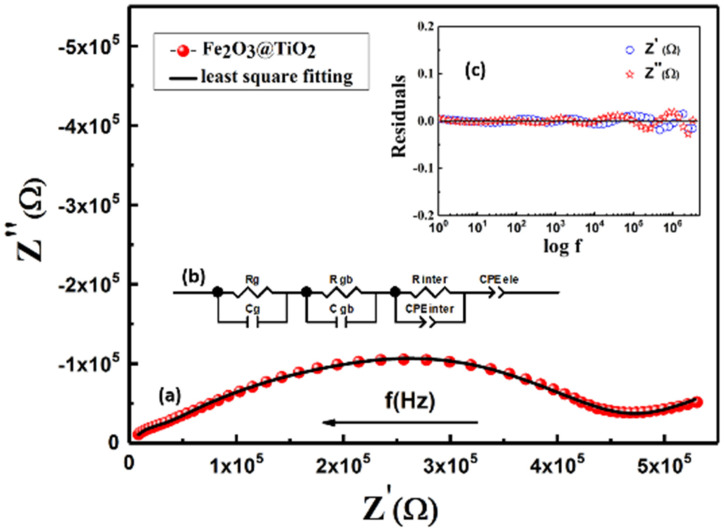
(**a**) Complex plain plot of Fe_2_O_3_@TiO_2_ sample prepared from Ginger (**b**) equivalent circuit obtain from ZView fitting (**c**) residue obtain for Z′ (Ω) and Z″ (Ω) from ZView fitting.

Impedance spectroscopy is a convenient technique that helps in the study of different electroactive regions in a polycrystalline ceramic. Figure 5a shows the complex plain plot of the as-prepared α-Fe_2_O_3_@TiO_2_. The frequency increases from right to left, as the arrow indicates in Figure 5a. The plot comprises of a depressed semicircle with a spike at the lower frequency side. The amount by which the center of the semi-circular arc was displaced below the real axis is measured in terms of the depression angle θ. It is an important parameter to judge the presence of distributed elements (heterogeneity and defects) in the system, which results in more than one relaxation phenomena. The resultant arc gets distorted by the multiple relaxation processes, holding time constant, within two orders of magnitude or less [34,35]. Thus, the relaxation time, rather than having a single value, is distributed over a range of frequencies. The depression angle estimated by the ZView software was found to be 45.208°. Considering the high value of θ, the constant phase element (CPE) was used in place of a capacitor to consider the non-ideal capacitive behavior. CPE and Care were related by the following expression:

C = CPE1/n R(1 − n)/n(2)

where R is the resistance of the cell, and n is the measure of deviation from ideal behaviour; n = 0 indicates purely resistive behaviour, and n = 1 indicates purely capacitive behavior.

Sintered polycrystalline materials consisted of grains, grain boundaries (mostly containing segregated impurities & pores), as well as surface layer and sample electrode interfaces [36]. The high-frequency response can usually be attributed to the bulk of polycrystalline material, whereas comparatively intermediate- and low-frequency responses may represent the electrical properties of electroactive regions like highly resistive grain boundaries, as well as surface layer and electrode resistances [35,37].

The ZView software was employed to find the electrical parameters, i.e., capacitances and resistances of respective electroactive regions by finding the best fitting model for the impedance plane plots of the Fe_2_O_3_@TiO_2_ sample. An equivalent circuit model obtained from the fitting is shown in Figure 5b, containing (Rg Cg) (Rgb CPEgb) (Rsurface CPEsurface) (CPEe) cells. The order of magnitude of capacitance associated with various electroactive regions has been well reported in the literature [38]. By comparing the values of capacitance obtained from the ZView fitting, (shown in Table 2) with already published literature [38], it can be assumed that Rg Cg and Rgb CPEgb cells are associated with grain and grain boundaries, respectively, i.e., Cg~10^−12^ and Cgb~10^−10^ (equivalent capacitance is calculated from Equation (2)), whereas Rsurface CPEsurface gives information about ongoing surface phenomenon, i.e., equivalent capacitance Csurface~10^−9^. This cell was a clear indicator of the presence of an active surface. It suggests that the layer was being formed by environmental gases, i.e., CO_2_ and O_2_ on the exposed pellet surface (not covered with silver paint) [27,31]. The low values of n surface & ne are also an indicator of the surface diffusion/adsorption from the surface, thus resulting in the high resistance (R surface = 4.02 × 10^4^ Ω), where CPEe represents electrodes as Ce~10^−6^. To validate the fitted model, residue was plotted against the frequency (shown in inset Figure 5c). It should be noted that the difference between the experimental and fitted value of Z′ and Z″ is approximately zero for a considerable range of the frequency. However, the inductance introduced by 1 m coaxial cables contributed to deviation at high frequencies, which is known to occur in the impedance analyzers at the higher ends of frequency spectrums [36]. Furthermore, the Chi-square value and sum of squares for the fitted model was found to be 2 × 10^−4^ and 0.024, respectively, which is indicates good fit.

Generally, in ceramics, grain boundaries are more resistive than grains. Decreased sintering temperatures and slow diffusion of ions results in chemical microheterogeneity [39]. The reoxidation is mainly limited to grain boundaries. This makes grain boundaries more resistive compared to the grains [40,41]. Furthermore, the non-stoichiometric distribution of oxygen and the presence of the carrier’s traps in grain boundaries form a barrier layer that hinders the carrier’s transport throughout the sample. In addition to higher resistance, this thin barrier layer possesses higher capacitance as inverse proportionality exists between the thickness, d, and the capacitance, C, (C ∝1/d) [42]. Because of such values of electrical parameters, the response of grain boundaries occurs at a lower frequency spectrum compared to grains. The values of R and C (or CPE) for grain and grain boundaries can be compared in Table 2. The resistance of grain boundaries is larger than the grains’ resistance which indicates the agglomeration of particles, as suggested from the above SEM images (Figure 3a) [43].

In composite materials, a depletion layer appears due to the difference in work function of the two materials comprising the composite. Also, non-stoichiometry and defect formation near the sample surface may lead to the formation of similarly capacitive surface layers. It has been previously reported that oxygen-deficient surfaces are prone to chemisorption of CO_2_ and O_2_, leading to the formation of a carbonate layer on the surface of pellets [36]. This relatively thin surface layer, indicated by Rsurface Csurface, acts as a high capacitance in parallel with a large resistor. High porosity, seen in the SEM image, suggests an active surface of the sample, which is highly reactive towards environmental effects. Moreover, the FTIR spectrum showed the presence of C–O vibration stretching, suggesting the presence of carbonate layers on the composite surface.

Spikes observed (i.e., at low frequencies in the sample) can be attributed to blocking capacitance effects at the crystal-electrode interface, i.e., oxide-ion conduction in the sample [44]. Electrode interfaces between the surfaces of pellets are sensitive to the heterogeneity of the polycrystalline material. If the medium is heterogeneous, the electrode interfaces are lessened and outweighed by the large number of grain boundaries between the electrodes. Only the CPE component is used to model the electrode sample interface, indicating minimum resistance offered by this region compared to others [43].

## 5. Conclusions

Heterogeneities in nanocomposite Fe_2_O_3_@TiO_2_ mixed-metal oxide powders were discussed, having been successfully prepared by green methods using ginger roots in a short time at a low calcination temperature. The synthesized samples were characterized using various analytical techniques. XRD and FTIR confirmed the formation of the Fe_2_O_3_@TiO_2_ composite in both samples. While the SEM indicated the formation of the nanocomposite, with sizes varying from 100 nm to 200 nm, DLS confirmed the agglomeration of particles. FTIR indicated the adsorption of CO_2_ gas on the surface of the sample. Electrochemical impedance spectroscopy (EIS) was performed at room temperature. Complex plain plot, equivalent circuit, and ZView fitting data confirmed the co-existence of grain, grain boundaries, and the presence of a carbonate layer on the surface that registered a separate and prominent relaxation phenomenon at the lower end of the frequency spectrum.

## Figures and Tables

**Table 1:** Elemental composition details as obtained from EDX.

Element	Weight %	Atomic %	σ
Fe	63.5	33.33	0.6
O	33.2	63.64	0.6
Ti	3.3	3.03	0.2
Total	100%	100%	

**Table 2:** Resistance and capacitance value for every component obtained from ZView fitting.

Fitting Parameters	Fe_2_O_3_@TiO_2_
Rg (Ω)	1.1 × 10^4^
Rgb (Ω)	4.03 × 10^5^
Rsurface (Ω)	4.02 × 10^4^
Cg (F)	14.5 × 10^−12^
Cgb (F)	1.45 × 10^−10^
Csurface (F)	2.95 × 10^−9^
Ce (F)	4.8 × 10^−6^
nsurface	0.50923
ne	0.36214

## Data Availability

Not applicable.

## References

[1] Padmanabhan N.T., Thomas N., Louis J., Methew D.T., Ganguly P., Pillai S.C. (2021). Graphene coupled TiO_2_ Photocatalysts for environmental applications: A review. Chemosphere.

[2] Serga V., Burve R., Krumina A., Romanova M., Kotomin E.A., Popov A.I. (2021). Extraction–Pyrolytic Method for TiO_2_ Polymorphs Production. Crystals.

[3] Verchere A., Cottrino S., Fantozzi G., Mishra S., Gaudisson T., Blanchard N., Pailhes S., Daniele S., Floch S.L. (2020). Effect of High Pressure Spark Plasma Sintering on the Densification of a Nb-Doped TiO_2_ Nanopowder. Ceramics.

[4] Kang X., Liu S., Dai Z., He Y., Song X., Tan Z. (2019). Titanium Dioxide: From Engineering to Applications. Catalysts.

[5] Zhou Z., Yin H., Zhao Y., Zhang J., Li Y., Yuan J., Tang J., Wang F. (2021). Synthesis of Magnetic α-Fe_2_O_3_/Rutile TiO_2_ Hollow Spheres for Visible-Light Photocatalytic Activity. Catalysts.

[6] Liu J., Xu J., Liu Z., Liu X., Che R. (2014). Hierarchical magnetic core-shell nanostructures for microwave absorption: Synthesis, microstructure and property studies. Sci. China Chem..

[7] Khan H., Swati I. (2016). Fe^3+^-doped Anatase TiO_2_ with d-d Transition, Oxygen Vacancies and Ti^3+^ Centers: Synthesis, Characterization, UV-vis Photocatalytic and Mechanistic Studies. Ind. Eng. Chem. Res..

[8] Wang Y., Jie W., Yang C., Wei X., Hao J. (2019). Colossal Permittivity Materials as Superior Dielectrics for Diverse Applications. Adv. Funct. Mater..

[9] Afqir M., Tachafine A., Fasquelle D., Elaatmani M., Carru J.-C., Zegzouti A., El Hammioui M. (2018). Effect of the synthesis route on the structural and dielectric properties of SrBi_1.8_Y_0.2_Nb_2_O_9_ ceramics. Int. J. Miner. Metall. Mater..

[10] Moniz S.J.A., Shevlin S.A., An X., Guo Z.-X., Tang J. (2014). Fe_2_O_3_–TiO_2_ Nanocomposites for Enhanced Charge Separation and Photocatalytic Activity. Chem.-Eur. J..

[11] Forster M., Potter R.J., Ling Y., Yang Y., Klug D.R., Li Y., Cowan A.J. (2015). Oxygen Deficient α-Fe_2_O_3_ Photoelectrodes: A Balance Between Enhanced Electrical Properties and Trap-Mediated Losses. Chem. Sci..

[12] Wang J., Gao Z., Li Z., Wang B., Yan Y., Liu Q., Mann T., Zhang M., Jiang Z. (2011). Green synthesis of graphene nanosheets/ZnO composites and electrochemical properties. J. Solid State Chem..

[13] Mindru I., Gingasu D., Culita D.C., Marinescu G., Patron L. (2014). Magnetic ferrites: Design and synthesis. Dekker Encyclopedia of Nanoscience and Nanotechnology.

[14] Philip D. (2010). Green synthesis of gold and silver nanoparticles using Hibiscus rosa sinensis. Phys. E Low-Dimens. Syst. Nanostruct..

[15] Manikandan A., Sridhar R., Antony S.A., Ramakrishna S. (2014). A simple aloe vera plant-extracted microwave and conventional combustion synthesis: Morphological, optical, magnetic and catalytic properties of CoFe_2_O_4_ nanostructures. J. Mol. Struct..

[16] Phumying S., Labuayai S., Swatsitang E., Amornkitbamrung V., Maensiri S. (2013). Nanocrystalline spinel ferrite (MFe_2_O_4_, M = Ni, Co, Mn, Mg, Zn) powders prepared by a simple aloe vera plant-extracted solution hydrothermal route. Mater. Res. Bull..

[17] Varma R.S. (2012). Greener approach to nanomaterials and their sustainable applications. Curr. Opin. Chem. Eng..

[18] Mohammadzadeh V., Barani M., Amiri M.S., Yazdi M.E.T., Hassanisaadi M., Rahdar A., Varma R.S. (2022). Applications of plant based nanoparticles in nanomedicine: A revew. Sustain. Chem. Pharm..

[19] Yang N., Li F., Jian T., Liu C., Sun H., Wang L., Xu H. (2017). Biogenic synthesis of silver nanoparticles using ginger (*Zingiber officinale*) extract and their antibacterial properties against aquatic pathogens. Acta Oceanol. Sin..

[20] Mohammadinejad R., Karimi S., Iravani S., Vaerma R.S. (2016). Plant derived nanostructures: Types and applications. Green Chem..

[21] Reda M., Ashames A., Edis Z., Bloukh S., Bhandare R., Abu Sara H. (2019). Green Synthesis of Potent Antimicrobial Silver Nanoparticles Using Different Plant Extracts and Their Mixtures. Processes.

[22] Das K., Bandyopadhyay T.K. (2004). Effect of form of carbon on the microstructure of in situ synthesized TiC-reinforced iron-based composite. Mater. Lett..

[23] Alias R. (2012). The Effects of Sintering Temperature Variations on Microstructure Changes of LTCC Substrate. Sintering of Ceramics–New Emerging Techniques.

[24] Saikumari N., Dev S.M., Dev S.A. (2021). Effect of calcination temperature on the properties and applications of bio extract mediated titania nano particles. Sci. Rep..

[25] Carvalho O.Q., Adiga P., Murthy S.K., Fulton J.L., Gutiérrez O.Y., Stoerzinger K.A. (2020). Understanding the Role of Surface Heterogeneities in Electrosynthesis Reactions. iScience.

[26] Abouzari M.S., Berkemeier F., Schmitz G., Wilmer D. (2009). On the physical interpretation of constant phase elements. Solid State Ion..

[27] Djuric Z.Z., Aleksic O.S., Nikolic M.V., Labus N., Radovanovic M., Lukovic M.D. (2014). Structural and electrical properties of sintered Fe_2_O_3_/TiO_2_ nanopowder mixtures. Ceram. Int..

[28] Li L., Yan J., Wang T., Zhao Z.-J., Zhang J., Gong J., Guan N. (2015). Sub-10 nm rutile titanium dioxide nanoparticles for efficient visible-light-driven photocatalytic hydrogen production. Nat. Commun..

[29] Zhang H., Wang Y., Wang H., Huo D. (2022). Room-temperature magnetoresistive and magnetocaloric effect in La1−xBaxMnO_3_ compounds: Role of Griffiths phase with ferromagnetic metal cluster above Curie temperature. J. Appl. Phys..

[30] Singh J., Sharma S., Basu S. (2019). Synthesis of Fe_2_O_3_/TiO_2_ monoliths for the enhanced degradation of industrial dye and pesticide via photo-Fenton catalysis. J. Photochem. Photobiol. A Chem..

[31] Kumar M.R., Abebe B., Nagaswarupa H.P., Murthy H.C., Ravikumar C.R., Sabir F.K. (2020). Enhanced photocatalytic and electrochemical performance of TiO_2_-Fe_2_O_3_ nanocomposite: Its applications in dye decolorization and as supercapacitors. Sci. Rep..

[32] Freund H.J., Roberts M.W. (1996). Surface chemistry of carbon dioxide. Surf. Sci. Rep..

[33] Chiorino A., Manzoli M., Menegazzo F., Signoretto M., Vindigni F., Pinna F., Boccuzzi F. (2009). New insight on the nature of catalytically active gold sites: Quantitative CO chemisorption data and analysis of FTIR spectra of adsorbed CO and of isotopic mixtures. J. Catal..

[34] Macdonald J. (1989). Impedance Spectroscopy: Emphasizing Solid Materials and Systems. Appl. Opt..

[35] Barsukov Y., Macdonald J. (2005). Impedance Spectroscopy: Theory, Experiment, and Applications.

[36] Muccillo R. (2009). Impedance spectroscopy analysis of zirconia:8 mol% yttria solid electrolytes with graphite pore former. J. Mater. Res..

[37] Akiya M., Nakamura H. (1986). Low ohmic contact to silicon with a magnesium/aluminum layered metallization. J. Appl. Phys..

[38] Irvine J.T.S., Sinclair D.C., West A.R. (1990). Electroceramics: Characterization by Impedance Spectroscopy. Adv. Mater..

[39] Jaiswal N., Kumar D., Upadhyay S., Parkash O. (2016). High electrical conductivity of nanocomposites based on Ce_0.82_Sm_0.16_Sr_0.02_O_1.90_ and (Li/Na)_2_CO_3_ for low temperature solid oxide fuel cells. Ceram. Int..

[40] Upadhyay S., Kumar D., Parkash O.M. (1996). Effect of composition on dielectric and electrical properties of the Sr_1−x_La_x_Ti_1−x_Co_x_O_3_ system. Bull. Mater. Sci..

[41] Idrees M., Nadeem M., Atif M., Siddique M., Mehmood M., Hassan M. (2011). Origin of colossal dielectric response in LaFeO_3_. Acta Mater..

[42] Liu J., Duan C.G., Yin W.G., Mei W.N., Smith R.W., Hardy J.R. (2004). Large dielectric constant and Maxwell-Wagner relaxation in Bi_2/3_Cu_3_Ti_4_O_12_. Phys. Rev. B.

[43] Prasad N.V., Srinivas K., Kumar G.S., James A.R. (2001). Impedance Measurements on TiO_2_–Fe_2_O_3_ Thin Films. Appl. Phys. A-Mater. Sci. Process..

[44] Vendrell X., West A. (2018). Electrical Properties of Yttria-Stabilized Zirconia, YSZ Single Crystal: Local AC and Long Range DC Conduction. J. Electrochem. Soc..

